# Aging under Pressure: The Roles of Reactive Oxygen and Nitrogen Species (RONS) Production and Aging Skeletal Muscle in Endothelial Function and Hypertension—From Biological Processes to Potential Interventions

**DOI:** 10.3390/antiox10081247

**Published:** 2021-08-04

**Authors:** Hollie Speer, Andrew J. McKune

**Affiliations:** 1 Faculty of Science and Technology, School of Science, University of Canberra, Bruce, ACT 2617, Australia; 2 Faculty of Health, School of Rehabilitation and Exercise Sciences, University of Canberra, Bruce, ACT 2617, Australia; Andrew.McKune@canberra.edu.au; 3 Research Institute for Sport and Exercise (UC-RISE), University of Canberra, Bruce, ACT 2617, Australia; 4 Discipline of Biokinetics, Exercise and Leisure Sciences, School of Health Science, University of KwaZulu-Natal, Durban 4000, South Africa

**Keywords:** aging, endothelial dysfunction, hypertension, inflammation, oxidative stress

## Abstract

The proportion of adults living with hypertension increases significantly with advancing age. It is therefore important to consider how health and vitality can be maintained by the aging population until end of life. A primary risk factor for the progression of cardiovascular diseases (CVD) is hypertension, so exploring the factors and processes central to this burden of disease is essential for healthy aging. A loss of skeletal muscle quantity and quality is characteristic in normal aging, with a reduction of vasodilatory capacity caused by endothelial dysfunction, and subsequent increase in peripheral resistance and risk for hypertension. Reactive Oxygen and Nitrogen Species (RONS) encompass the reactive derivatives of NO and superoxide, which are continuously generated in contracting skeletal muscle and are essential mediators for cellular metabolism. They act together as intra and intercellular messengers, gene expression regulators, and induce programmed cell death. In excessive amounts RONS can inflict damage to endothelial and skeletal muscle cells, alter signaling pathways or prematurely promote stress responses and potentially speed up the aging process. The age-related increase in RONS by skeletal muscle and endothelial mitochondria leads to impaired production of NO, resulting in vascular changes and endothelial dysfunction. Changes in vascular morphology is an early occurrence in the etiology of CVDs and, while this is also a normal characteristic of aging, whether it is a cause or a consequence of aging in hypertension remains unclear. This review serves to focus on the roles and mechanisms of biological processes central to hypertension and CVD, with a specific focus on the effects of aging muscle and RONS production, as well as the influence of established and more novel interventions to mediate the increasing risk for hypertension and CVD and improve health outcomes as we age.

## 1. Introduction

By 2025, the number of people aged 65 years and above will overtake the number of children aged between 0–14 years [1]. By 2050, almost one quarter of all Australians will be 65 years or older [1]. It is therefore critical to consider how health and vitality can be maintained by this majority until end of life. The incidence of cardiovascular disease (CVD) increases with advancing age and is a leading cause of death worldwide, placing an unprecedented health care burden on the global population [1,2]. A primary risk factor for the progression of CVD is hypertension, which also increases more rapidly in prevalence after the age of 60 years, and for this reason is generally considered an aging disorder [3]. Detrimental health effects increase in preponderance and severity as blood pressure rises, harboring extensive systemic pathophysiological consequences such as increased peripheral resistance, vessel damage, and if untreated or poorly managed, can result in a significant increase in all-cause mortality [4].

**Figure 1:**
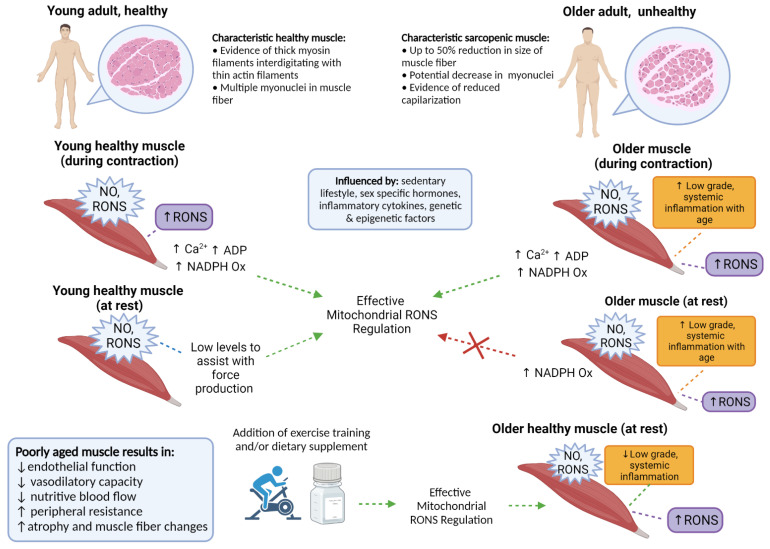
Comparison of characteristically healthy and sarcopenic muscle, in young and older adults. Essential RONS signaling is apparent in young healthy muscle during contraction but becomes greater with advancing age at rest. Effective and proportionate regulation of RONS could be influenced by several modifiable and non-modifiable factors, with the capacity for self-regulation becoming less efficient with age. The lower half of the figure indicates the consequences of poorly aged muscle, with specific interventions supporting healthy muscle aging and RONS regulation, even in the presence of increased RONS production. Created with BioRender.com.

Reactive Oxygen and Nitrogen Species (RONS) encompass the reactive derivatives of Nitric Oxide (NO) and superoxide [5]. RONS are continuously generated in contracting skeletal muscle, and play an important role in the regulation of many cellular and physiological processes [5]. Often termed “free radicals”, in *acute* instances they act together as essential intra and intercellular messengers, but excessive or *chronic* exposure can inflict damage, alter cellular communication pathways, and promote states of “oxidative stress”—thereby influencing muscle and vessel physiology (Figure 1) [5,6]. Furthermore, skeletal muscle specific-RONS play roles in exercise and muscular adaptations and are essential in mediating many metabolic responses [7]. The point at which these effects are no longer useful or become detrimental is still an interesting topic of discussion, and we hypothesize this depends on the degree of chronic vs. acute incidences of exposure to oxidative stress throughout life. While the associations of hypertension with body composition, specifically overweight and obesity, are relatively clear, there is still little known or validated about the link between RONS and endothelial dysfunction with age-related hypertension and aging skeletal muscle. This should be a specific focus due to the relationship between increased body fatness and increased risk for sarcopenia with age [8].

The effects of RONS produced by aging muscle on cellular and epigenetic processes [6], as well as the cells associated with endothelial function and vessel stiffness [9], and the modifiable effects of diet and exercise [10] may have an interrelated influence on the molecular mechanisms for blood pressure regulation, vessel and muscle repair pathways, and gene expression. This review aims to explore the biological processes central to vascular changes with aging and the link between skeletal muscle RONS production with advancing age. There is an additional focus on established interventions such as exercise, as well as newer and more novel dietary interventions as potential mediators for hypertension risk, thereby increasing active life expectancy and contributing to healthy aging.

## 2. Discussion

### 2.1. Endothelial Dysfunction

The endothelium, amongst other properties, is responsible for regulating vascular tone—any disturbance to this is termed endothelial dysfunction, whereby impairments in vasorelaxation or vasoconstriction in response to traditional endothelium dependent dilators and constrictors can occur [11]. RONS are produced normally in cells during mitochondrial respiration and energy generation, functioning as cellular signaling molecules, and playing roles in gene regulation, and programmed cell death [12,13]. Thus, cells can maintain a steady state in which RONS are present at low concentrations but do not cause damage [13], and in reduced amounts, mitochondrial stress can lead to incremental health within a cell—a paradigm known as mitohormesis [14]. In the absence of homeostatic compensation, increased production, decreased clearance, or the accumulation of RONS leads to the activation of stress-sensitive signaling pathways, lipid peroxidation, and the synthesis of proteins that inflict cellular damage, and contribute to the vascular complications of hypertension, further compounding the effects of endothelial dysfunction and therefore peripheral resistance [9]. It is this increased production or decreased scavenging of RONS that can lead to an excess, or chronic exposure of free radicals, termed oxidative and nitrosative stress [15]. This has been implicated in a wide variety of pathologic processes, contributing to vascular stiffness and endothelial dysfunction, directly linked with hypertension [9,15]. Endothelial dysfunction occurs early in the course of vascular morphology, common to atherosclerosis and associated vascular diseases, including hypertension and diabetes [9,16]. Excessive RONS production is also considered one of the central hallmarks of aging [5,6]. However, under healthy conditions, RONS regulate vascular function through redox-sensitive signaling pathways, and all vascular cell types produce RONS primarily via membrane-associated Nicotinamide Adenine Dinucleotide Phosphate (NADPH) oxidases, as well as via mitochondria, influencing vascular function by modulation of contraction and dilation [11,17]. This can be considered beneficial with respects to vascular adaptations in response to normal physiological stimuli or with exercise, and some NADPH oxidases have been identified as mediators for endothelial dysfunction [18,19]. Even so, given the increase in sympathetic nerve activity in the muscle accompanying normal aging, alongside changes in sympathetic vessel tone, and thereby increased peripheral resistance both in the vessels and skeletal muscle [20], there remains a need to explore the change in beneficial and detrimental roles of RONS as we age. It is proposed that NADPH-generated RONS are higher in older persons as compared to younger individuals [21,22], and NADPH oxidase has been implicated as a source of chronic ROS production in skeletal muscle, linking NADPH oxidase-derived ROS to endothelial dysfunction [23].

### 2.2. Inflamm-Aging

In addition to skeletal muscle myocytes, RONS are produced by activated leukocytes, particularly neutrophils and macrophages, during inflammatory reactions [13,17]. Inflammation is a major component of the initiation and progression of most CVDs, a key mechanism of endothelial dysfunction and arterial damage [24]. Both are central to this, with RONS and inflammatory biomarkers indicative of risk of pathology as well as raised levels indicating the presence of endothelial dysfunction and damage [25]. As a result this has fostered the concept of ‘inflamm-aging’, a chronic low-grade inflammation that develops with advancing age [24]. Therefore, the use of inflammatory biomarkers including immune cell subtypes, cytokines, and proteins such as C-Reactive Protein, as indicators of allostatic load, or the “wear and tear” on physiological systems, are useful to identify the state of risk associated with vascular dysfunction and CVD [26,27]. With aging there is potential for an increased production of RONS from both myocytes and leukocytes associated with the “inflamm-aging” of aging [28].

### 2.3. Skeletal Muscle

In the context of the present review, a loss of skeletal muscle quantity and quality, termed sarcopenia, which occurs throughout the aging process has been linked with endothelial dysfunction and increased peripheral resistance [29]. As skeletal muscle is an important site of peripheral resistance, a reduction of vasodilatory capacity in this tissue increases the risk for hypertension, with excessive and detrimental myocyte RONS production playing a major role in this process [15,30]. In moderate levels, RONS regulate carbohydrate metabolism and are indirectly involved in fat metabolism through the NAD-dependent deacetylase sirtuin-3 protein (SIRT3) [5]. This mediated change of lipids, proteins, and DNA by RONS can play a significant role in mitochondrial function, remodeling and repair pathways, protein turnover, gene expression, or epigenetic regulation [5,30,31]. Thus, when exceeding moderate levels, there may be an accumulation of mitochondrial damage, preventing the regenerative capacity of healthy cells, leading to bioenergetic decline and premature cell death [32]. This is specifically relevant in the context of aging skeletal muscle, given the paradigm that RONS are needed for adequate cellular performance during healthy exercise [7], and acute incidences of oxidative stress are suggested to be protective and beneficial [33]—so the point at which this then becomes detrimental (during exercise and at rest) is up for debate. Skeletal muscle at rest encompasses relatively high vascular tone attributed to the partial constriction of arterioles of vascular smooth muscle in the vessels, and the innervation of surrounding sympathetic nerves, allowing for a basal level of force to be present pre-emptive to contractile activity [7,34]. In younger, healthy muscle, acute instances of RONS—due to the modification of intracellular redox balance—act as signaling molecules during skeletal muscle contraction [34]. Thereby inducing the activation of adaptation mechanisms which consist of structural and biochemical changes that can lead to protection against potential damage caused by further exercise, or repair cells as a result of functional modifications [34,35]. The subsequent changes in skeletal muscle size and strength with age see a severe blunting of these adaptation mechanisms, and the implications of RONS in this process is of highlighted interest [34,36]. Age-associated oxidative stress and the ensuing damage is speculated to be a main contributor to sarcopenia and muscle atrophy fundamental to aging [37]. A 2013 study in rodents has indicated that RONS are increased in skeletal muscle fibers at rest, but not during contraction, with suggestions that these changes are likely due to an increase in endogenous oxidant generation rather than a lack of RONS scavenging [36]. Age related overproduction of RONS can generate chronic states of oxidative stress, therefore damaging the muscle; but basal levels are required for regulation of intracellular signaling pathways, essential for exercise and healthy aging [33]. It may be that RONS become a “*necessary evil*” beyond a certain threshold or age, and investigations into the micro and macrovascular environments are needed to quantify the changes in skeletal muscle RONS production in younger and older (healthy and non-healthy) people.

### 2.4. Biological Aging

The regulation of cellular repair pathways, gene expression, and epigenetic mechanisms play a crucial role in healthy aging. Telomeres consist of repeated DNA sequences that create protective caps at the ends of chromosomes, protecting linear chromosome ends from damage that may ultimately lead to cellular death and adverse health [38]. Telomeres progressively shorten over the period of a lifetime with each cellular replication; however numerous studies have implicated the presence of excessive RONS in accelerated telomere shortening [31]. This telomere attrition is considered one of the central hallmarks of aging, as well as both a marker of cell aging and a causal factor in cell aging [6]. Short telomeres impair the functional ability for a cell to divide properly, and when DNA replication cannot appropriately occur, the cell either undergoes cell death, or functions poorly, triggering genomic instability in pre-malignant cells [38]. Another DNA-based biomarker that is altered with aging is methylation of cytosine residues of cytosine-phosphate-guanine dinucleotides (CpGs), referred to as DNA methylation [39]. DNA methylation (DNAm) plays a pivotal role in gene silencing, where the presence of multiple methylated CpG sites has the capacity to stably *“switch off”* genes [40]. Fundamentally, this has both advantageous and deleterious effects, ultimately augmenting the ways in which our bodies are programmed to be susceptible to disease [41]. The development of epigenetic clocks has utilized specific levels of CpG methylation to estimate biological age contrast to chronological age, to predict the time-to-onset of specific age-related diseases [39]. Senescent cells accumulate with age, secrete inflammatory cytokines, and have well-established roles in the promotion of degenerative diseases and pathology with aging [31,38]. While senescent cells can be produced as a byproduct of chronic oxidative stress [38], it is still unclear as to whether senescent cells also contribute to excessive RONS production. The reduced regenerative capacity or turnover of healthy cells in this state can result in mitochondrial dysfunction and DNA damage, further adding to the vicious cycle of inflammation and disease onset [31,38]. Studies investigating telomere length (TL) in human tissues [42], animal models [43] and cell cultures [44] have shown associations between shorter TL and various types of inflammatory and CVDs [45]. This information may be critical in determining clinical presenting symptoms as well as cellular symptoms of CVD and associated complications such as endothelial dysfunction. Telomere Length is also controlled by epigenetic modifications to telomeric material and information, and while TL may be a useful biomarker associated with biological age, the robustness of TL assessment itself remains varied and questionable [41,46,47]. Perhaps a combination of both TL and DNAm can form a composite biomarker panel to provide a clearer and more quantifiable measure for aging and susceptibility to diseases including hypertension [41,47]. Lu et al. [39] propose a DNAm-based estimator of TL in predicting measures of age-related pathologies such as physical fitness and age at menopause based on large cross-sectional study data. This gives rise to the hypothesis that preservation or maintenance of TL, in combination with favorable gene silencing, can contribute to delaying disease onset—for which this information could potentially reduce the risk for diseases associated with aging, and more specifically, hypertension [48]. However, the extent to which TL and DNAm are influenced by environmental and contributing lifestyle factors like diet and exercise is still a topic of discussion.

### 2.5. Physical Activity and Exercise Interventions

Inadequate physical activity is a worldwide public health problem, accounting for up to 10% of the global burden of major chronic non-communicable diseases [49]. Changes in skeletal muscle adaptations to RONS, as well as hemostatic and inflammatory variables have been inversely associated in a dose-dependent manner with physical activity [50]. An animal study by Laufs et al. [51] identified that physical inactivity increased vascular NADPH-oxidase expression and activity, enhancing vascular production of RONS in sedentary mice as opposed to those exposed to physical activity. The study suggests that physical inactivity, which is often coupled with visceral fat accumulation, as well as increased macrophage infiltration into adipose tissue and vascular lipid peroxidation, results in the release of inflammatory cytokines and consequently, superoxide, contributing to increased NADPH-oxidase, and the over production of RONS [51,52]. Systemic inflammation and RONS production, associated with physical inactivity and visceral fat accumulation, are also associated with age-related declines in NO production and availability in endothelial cells and skeletal myocytes [53]. Based on the findings from Palomero et al. [36], the presence of RONS within these cells are expected to be higher in older people, but we suspect this becomes gravely worse when coupled with physical inactivity.

Exercise training, particularly aerobic and resistance training, has been shown to improve NO-induced vasodilatation and decrease BP—these vaso-protective mechanisms of exercise training can play an important role in determining and identifying risk factors associated with hypertension and CVD [54,55,56]. Exercise has many positive and protective effects regarding overall health, and in some cases can aid in the prevention of all-cause mortality, as well as improve endothelial function, increase our innate antioxidant defenses, and reduce chronic inflammation [49,57]. However, care should be taken to identify potential sex differences and sex-specific hormone regulation regarding inflammatory and vascular response with exercise in post-menopausal populations, and the use of sex-specific interventions should be considered to effectively maintain or improve vascular health [58]. A 2018 study showed that all extremity exercise (high-intensity interval training and moderate intensity continuous training) reduced arterial stiffness in healthy older people aged 55–79 years [56]. While this is a positive healthy aging outcome, further research has suggested that specific exercise prescriptions may be required for individuals who have hypertension. A 2020 meta-analysis [59] assessed the effects of exercise training on ambulatory BP in individuals with hypertension (both treated and untreated), with interventions lasting between 8–24 weeks and inclusive of 3–5 sessions per week (~24–60 min/session). Aerobic exercise was shown to significantly reduce ambulatory BP in individuals who were taking antihypertensive medication (−4.9 mmHg SBP and −2.8 mmHg DBP), but negligible in those who were unmedicated compared to baseline [59]. However, habitual aerobic exercise has been shown to increase plasma concentrations of nitrite/nitrate and decrease BP in normotensive older people [60]. This was evident at a frequency of exercise ranging between 2.3–7 d/wk, for 33–110 min/d, at 59–91% of maximal heart rate. It may be that habitual aerobic exercise between these ranges throughout mid-life is an effective preventative approach, and as a complementary treatment in later life when paired with medication.

A 2020 study [61] assessing a six week aerobic exercise training program in young and older people improved mitochondrial function in the older individuals to the same extent as younger individuals from baseline, despite an age difference of more than 50 years between groups. After eight weeks of deconditioning, the training-induced increases in mitochondrial electron transport chain efficiency, decreased more rapidly in older people compared with the younger group [61]. This forms the basis that perhaps there is greater overall mitochondrial function and consequently lower resting RONS and inflammation in younger or healthy muscle. Regular exercise has been proposed to lower pro-inflammatory cytokines such as TNF-α and promote myokine (IL-6, IL-10) production through muscle contraction to induce anti-inflammatory responses [62]. With respects to myokines—the response to muscle contraction during exercise—IL-6 and IL-10 may balance and counteract the effects of pro-inflammatory adipokines such as leptin [53], where the associations between skeletal muscle, the cardiovascular system and adipose tissue have been identified to play a key role in the regulation of blood pressure and the development of hypertension [63]. Irisin, which is secreted from muscles in response to exercise, has been positively correlated with systolic BP and has been associated with hypertension-related stroke in humans [63]. As well as this, the hormone also plays part in lowering total BP and ameliorating endothelial dysfunction in rats via the AMPK-Akt-eNOS-NO signaling pathway [64]. These findings indicate that exercise can modulate endothelial function, ultimately increasing vessel health while also maintaining muscle mass and quality. This further supports exercise and physical activity as a nonpharmaceutical therapies in hypertension treatment and prevention.

### 2.6. Use of Dietary Antioxidants and Dietary Supplements

The idea of modifying the diet to enhance health can date back to the training of the first Olympic athletes [65] and is generally driven by the desire to optimize daily function or performance [66]. Generally speaking, in order for supplements to be effective, they must provide nutritional value that is deficient to cells, or exert a pharmacologic effect on cellular processes and responses [66]. Oftentimes, a limitation to many clinical studies assessing antioxidant supplementation or a dietary antioxidant intervention is the difficulty to observe and measure direct effects in target tissues such as the skeletal muscle or within the vessel wall [67], and so surrogate markers are used to describe a cause-and-effect relationship without sufficient validation [68]. Dietary antioxidants can include well-known vitamins such as Vitamins E and C; however, previous pharmacological studies, randomized control trials, and meta-analyses have shown that vitamin antioxidant therapy alone demonstrates minimal effect in humans, specifically with regards to improving cardiovascular health [69,70,71]. This now encourages the need for specifically targeted antioxidant compounds or dietary supplements, which may be beneficial in combating oxidative stress and increasing overall health with some antioxidants also exhibiting anti-inflammatory properties [72]. Increased levels of ketone bodies in cells, such as β-hydroxybutyrate, support survival during states of physiological stress by serving as alternative sources of ATP [73]. Under normal conditions these ketone bodies are produced in small quantities, but become elevated by high-intensity exercise, or through diet modification [74,75]. Outside of diet modification, which can often be restrictive and unsuitable for certain individuals (such as those with T1DM) the use of exogenous or endogenous ketone supplements consumed in addition to a regular diet has become an appealing alternative due to being less restrictive in nature [76,77]. A 2018 study [78] evaluated the effects of ketone bodies on endothelial cells and assessed DNA oxidative damage and the activation of a transcriptional factor involved in cellular responses to oxidative stress (Nrf2). Cells that were pre-treated with ketone bodies for 48hr were more resistant to subsequent oxidative insults, having significantly decreased DNA damage compared to control. These findings suggest that ketone bodies, by inducing moderate oxidative stress activate the transcription factor Nrf2, promote the transcription of target genes involved in the cellular antioxidant defense system [78]. The authors [78] noted that while these findings seem positive, this may not be protective with chronic or long-term exposure. This is due to the suppression of apoptosis, improvement of mitochondrial function and redirection of glucose metabolism toward NADPH generation, ultimately increasing the tendency for cellular proliferation—which may pose harmful effects in the long-term as a result of impaired oxidative cell status [78]. With this considered, in acute instances, the effects of ketones, specifically β-hydroxybutyrate, can induce adaptations similar to that of mitohormesis [79] but the suitability of this intervention should be assessed on a case by case basis, as it is not a “one size fits all” approach [77]. A more broadly suitable candidate could be a selective, targeted mitochondrial supplement as a blanket intervention for chronic oxidative stress and vessel function to combat aging RONS production and preserve DNA damage. A 2018 placebo controlled RCT investigating chronic (6 weeks) supplementation with a mitochondrial-targeted antioxidant—specifically synthesized ubiquinone (CoQ10, sold under the brand name *MitoQ*)—in humans demonstrated improved vascular function and decreased arterial stiffness in older men aged 60–79 years [80]. While circulating markers of inflammation were not different between groups, vascular endothelial function (as measured by NO-dependent brachial artery flow-mediated dilation) was statistically significant, being 42% higher following *MitoQ* supplementation vs. placebo. Additionally, a 2003 study [81] indicated that by minimizing the extent or exposure of oxidative stress through *MitoQ* supplementation, telomere shortening is significantly slowed. These findings suggest that therapeutic strategies specifically targeting the mitochondria or excessive mitochondrial RONS may hold promise in treating age-related vascular dysfunction, reducing BP, counteracting excessive RONS production, reducing chronic low-grade, systemic inflammation, oxidative stress and maintaining telomere length to improve clinical outcomes and healthy aging.

## 3. Conclusions

The current and rising prevalence of CV related diseases are a concern affecting people at any life stage, but particularly in later life. There is a need for research focusing on the interrelated cellular micro- and macro-vascular skeletal muscle environments, and further investigative research is needed to determine the feasibility of increasing endothelial function and decreasing detrimental RONS production with exercise training and/or readily available antioxidant or dietary supplementation. This review explored the yet to be established link between poor muscle quality and endothelial dysfunction, as well as increased or excessive skeletal muscle RONS production, with age-related hypertension. We put forward the concept that mitochondrial dysfunction and inflammation can result in oxidative DNA damage, accelerated telomere shortening and altered gene expression, ultimately rendering a volatile environment for the pathogenesis of hypertension and unfavorable health outcomes. These suggestions aim to find practical means of reversing vasodilatory decrements, thereby reducing the risk for and incidence of CV related diseases such as hypertension in older populations, resulting in increased quality of life and healthy aging.
